# CD137 (4-1BB) requires physically associated cIAPs for signal transduction and antitumor effects

**DOI:** 10.1126/sciadv.adf6692

**Published:** 2023-08-18

**Authors:** Javier Glez-Vaz, Arantza Azpilikueta, María C. Ochoa, Irene Olivera, Gabriel Gomis, Asunta Cirella, Carlos Luri-Rey, Maite Álvarez, Jose L. Pérez-Gracia, Sergio Ciordia, Iñaki Eguren-Santamaria, Raluca Alexandru, Pedro Berraondo, Carlos de Andrea, Álvaro Teijeira, Fernando Corrales, Juan M. Zapata, Ignacio Melero

**Affiliations:** ^1^ Program of Immunology and Immunotherapy, Cima Universidad de Navarra, Pamplona, Spain.; ^2^ Navarra Institute for Health Research (IDISNA), Pamplona, Spain.; ^3^ Departments of Immunology-Immunotherapy, Pathology and Oncology, Clínica Universidad de Navarra, Pamplona, Spain.; ^4^ Centro de Investigación Biomédica en Red de Cáncer (CIBERONC), Madrid, Spain.; ^5^ Functional Proteomics Laboratory, CNB-CSIC, Proteored-ISCIII, Madrid, Spain.; ^6^ Instituto de Investigaciones Biomédicas Alberto Sols (IIBm), CSIC-UAM, Madrid, Spain.; ^7^ Instituto de Investigación Sanitaria La Paz (IdiPaz), Madrid, Spain.; ^8^ Nuffield Department of Medicine, University of Oxford, Oxford, UK.

## Abstract

CD137 (4-1BB) is a member of the TNFR family that mediates potent T cell costimulatory signals upon ligation by CD137L or agonist monoclonal antibodies (mAbs). CD137 agonists attain immunotherapeutic antitumor effects in cancer mouse models, and multiple agents of this kind are undergoing clinical trials. We show that cIAP1 and cIAP2 are physically associated with the CD137 signaling complex. Moreover, cIAPs are required for CD137 signaling toward the NF-κB and MAPK pathways and for costimulation of human and mouse T lymphocytes. Functional evidence was substantiated with SMAC mimetics that trigger cIAP degradation and by transfecting cIAP dominant-negative variants. Antitumor effects of agonist anti-CD137 mAbs are critically dependent on the integrity of cIAPs in cancer mouse models, and cIAPs are also required for signaling from CARs encompassing CD137’s cytoplasmic tail.

## Teaser

cIAPs are physically associated to CD137 (4-1BB) and required for the functions of this T-cell costimulatory receptor.

## INTRODUCTION

CD137 (4-1BB, TNFRSF9) is arguably one of the most potent costimulatory molecules on T and natural killer (NK) lymphocytes (*1*, *2*). CD137 expression on T lymphocytes is not constitutive and requires antigen recognition to be induced (*3*). Once on the plasma membrane, if ligated and crosslinked by its natural ligand (CD137L, 4-1BBL) or agonist antibodies, CD137 conveys signals that costimulate several T cell functions (*4*–*7*) and confer protection from programmed cell death (*8*, *9*).

CD137 is currently one of the most attractive molecular targets for antitumor immunotherapy (*10*, *11*). In mice, administration of CD137 agonists to tumor-bearing mice increases cytotoxic T cell responses against tumor antigens that are curative in a number of instances (*12*). In humans, urelumab was the first agonist anti-CD137 monoclonal antibody (mAb) evaluated in clinical trials. Urelumab development was halted because of causing severe hepatitis in 10 to 15% of the patients in phase 2 clinical trials, as a result of an on-target effect at doses higher than 0.3 mg/kg (*13*, *14*). However, it showed that targeting CD137 in patients to enforce the antitumor activity of T cells was feasible and clinically active (*13*, *15*, *16*). Since then, another fully human anti-CD137 mAb, utomilumab (*17*, *18*), and several other anti-CD137–based immunotherapeutic agents, including mono-, bi-, and multi-specific antibodies, are being clinically developed (*19*–*25*). In addition, several chimeric antigen receptors (CARs) are designed to encompass the cytosolic tail of CD137 to deliver costimulatory signals when expressed in T and NK cells (*26*, *27*).

Despite the relevance of CD137 in immunotherapy, knowledge of the proteins involved in CD137 signaling is still incomplete. CD137 belongs to the tumor necrosis factor receptor (TNFR) family and shares many biochemical features with this protein subfamily in terms of its signaling machinery. Lacking a death domain and any known intrinsic enzymatic activity, CD137 reportedly relies for signaling on its association with TRAF2 (*28*, *29*), TRAF1 (*30*), and TRAF3 (*31*) as signal transduction adaptors. More downstream, CD137 signaling involves nuclear factor κB1 (NF-κB1) and NF-κB2 (*32*, *33*) as well as the activation of several mitogen-activated protein kinases (MAPKs) (*34*–*36*).

The early signaling events are proposed to be elicited by CD137 trimerization by ligand on the plasma membrane without published evidence for any conformational changes to be involved in signaling. CD137:CD137L trimers form a windmill-like structure, which would be the basic CD137 signaling unit, although the formation of a lattice of activated trimers has been proposed to account for more efficient CD137 signaling (*37*). TRAFs are the first molecules that are recruited to the activated TNFRs. It is unclear whether TRAFs could bind to nonstimulated CD137, but upon CD137 activation by ligand or agonistic antibody binding, a recruitment of TRAFs to the receptor occurs. TRAFs work as scaffold proteins to build up the CD137 signalosome. TRAF2 and TRAF3, but not TRAF1, are E3 ubiquitin ligases. TRAF2 can K63-polyubiquitinate itself and other substrates as a reportedly key event in CD137 signaling (*38*). Sphingosine-1-phosphate is a key cofactor for the E3 ubiquitin ligase enzymatic activity of TRAF2 (*39*).

Soon after CD137 engagement, the CD137 complexes are readily endocytosed, in a process that is dependent on TRAF2 and K63 polyubiquitination (*38*). On the basis of our knowledge on the signalosomes of other TNFR family members and on the basis of functional assays, we can speculate what other proteins might participate in CD137 signaling (*37*).

Among the proteins that might associate to the activated CD137 cytoplasmic tail and regulate its function are cIAP1 and cIAP2. A clue to the identity of these alternative E3 ubiquitin ligases in the signaling from TNFR members came from the studies on the TNFR1 complex that recruits cIAP1 and cIAP2 via TRAF2 to elicit what constitutes a potent antiapoptotic pathway (*40*). cIAP1 and cIAP2 are two homolog proteins involved in antiapoptotic signals and tumor biology, which belong to a family of proteins that mediates antiapoptotic functions (*41*). Although they contain a caspase binding domain, direct inhibition of caspases by cIAPs has not been substantiated (*42*). Crystal structures of cIAP2 bound to TRAF2 homotrimers and to (TRAF2)_2_TRAF1 heterotrimers proved the tight association among these proteins (*43*). Moreover, functional evidence for a role of cIAPs in CD137 signaling came from mice transgenic for a dominant-negative (DN) variant of cIAP2 with a mutated RING domain (*44*) that functionally outcompetes endogenous cIAP1 and cIAP2. In such cIAP2 DN transgenic mice, CD137 costimulation of T cells was found to be hampered (*44*).

cIAPs are rapidly degraded in the cell if the mitochondrial protein SMAC/DIABLO is released to the cytosol upon proapoptotic insults (*45*). The mechanisms involve the K48 polyubiquitination by cIAPs themselves. Such a function can be pharmacologically imitated by a family of compounds known as SMAC mimetics (SMCs), which effectively and rapidly deplete cIAPs from the cell in a proteasome-dependent manner (*46*).

Here, we have performed a proteomic approach to identify components of the CD137 signalosome. We have confirmed the physical association of cIAP1 and cIAP2 to the CD137 signaling complex. Moreover, we demonstrate that the functional presence of cIAPs in T lymphocytes is required for CD137 signaling, costimulation, and antitumor activity.

## RESULTS

### Identification of components of the CD137 signalosome

**Fig. 1.**
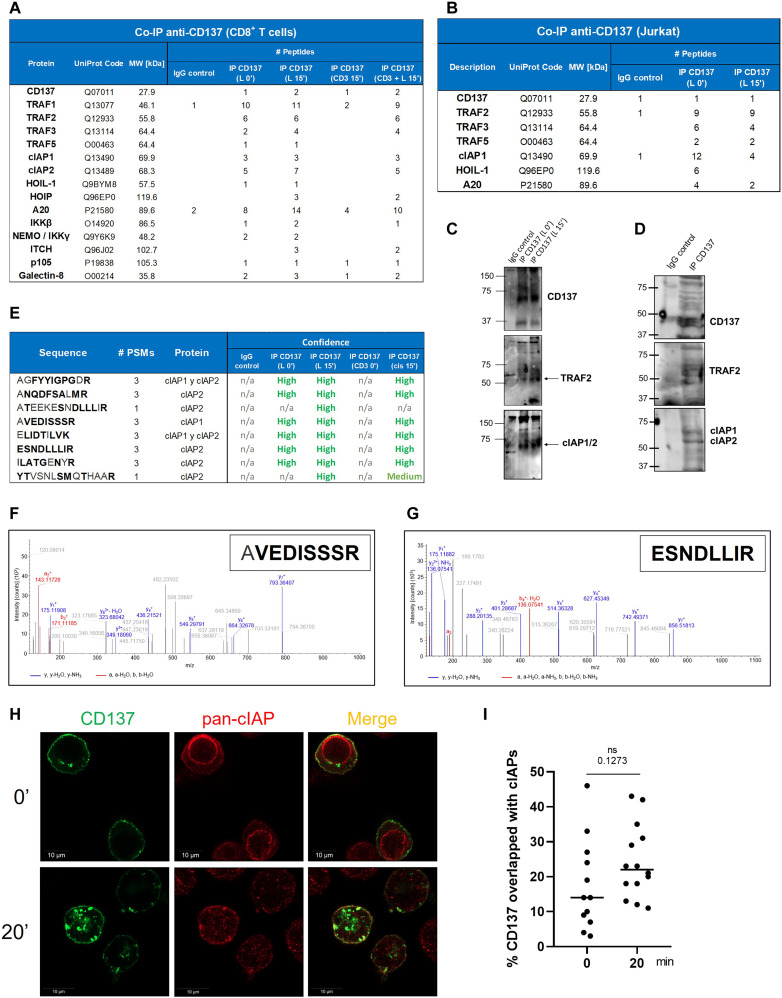
Signaling proteins physically associated with CD137. (**A**) Brij96 lysates from primary human CD8^+^ T cells from peripheral blood were immunoprecipitated (IP) with anti-CD137 covalently linked to magnetic microbeads. Tryptic peptides of pulled-down proteins were resolved. The list shows the proteins identified related to CD137 signaling and the corresponding accession number, and the molecular weights (MW) of the proteins are provided. Lysates were obtained from resting cells and cells cultured for 15 min on culture plates coated with CD3, CD137L, or both and compared in terms of the number of tryptic peptides from the proteins recovered in each condition. (**B**) Similar experiments as in (A) but with lysates from CD137 stably transfected Jurkat cells. (**C** and **D**) Immunoblots on the Jurkat immunoprecipitates revealed with the indicated specific anti-CD137, anti-TRAF2, and anti-cIAPs mAbs under nonreducing and reducing conditions. Immunoblot experiments were repeated at least three times with comparable results. (**E**) Details of the different cIAP1 and cIAP2 peptides identified by mass spectrometry in samples from (D). (**F** and **G**) Mass spectrometry patterns concluding the presence of the peptides AVEDISSR and ESNDLLIR that unequivocally denote the presence of cIAP1 and cIAP2 in the immunoprecipitates, respectively. (**H**) Confocal microscopy images of hCD137-GFP transiently transfected 293T cells that were permeabilized and stained with an anti–pan-cIAP mAb. Cells were examined at baseline or following 20 min culture in the presence of the anti-hCD137 6B4 mAb. (**I**) Quantification of the CD137:cIAP overlap in stained cells as in (H).

The composition of the proteins that constitute the signalosome of CD137 is only partially known. To get new insights into the proteins participating in early CD137-mediated signaling, we studied what proteins could be found physically associated with CD137 in activated primary human CD8 T cells. Such T lymphocytes were preactivated with microbeads coupled to anti-CD3 + anti-CD28 mAbs and interleukin-2 (IL-2) to induce intense CD137 surface expression and were subsequently rested in clean culture medium for 4 hours. CD8 T cell cultures were stimulated with plate-bound CD137L, anti-CD3, or both for 15 min at 37°C, and a certain part of the cells were left on ice (fig. S1A). Cells were then lysed in a buffer containing 1% Brij96 as a gentle detergent to preserve protein-to-protein associations as much as possible in the lysates. Immunoprecipitation was performed with magnetic microbeads covalently coated with the 6B4 anti-CD137 mAb (*38*) or control immunoglobulin G (IgG) mAb (fig. S1A). The obtained immunoprecipitates were digested with trypsin and subjected to high-performance liquid chromatography (HPLC) and mass spectrometry analyses to identify polypeptides that coprecipitated with CD137. Proteins that were enriched in the immunoprecipitates at 37°C were selected. Figure 1A shows the list of TNFR signal-related proteins found in the immunoprecipitates, and data S1 provides all the identified proteins.

As it should be expected, peptides for both CD137 and CD137L (used for CD137 activation in our experimental setting) were readily found in the CD137 immunoprecipitates (Fig. 1 and data S1 and S2). Among the other molecules that were found associated, the interaction with TRAFs was particularly relevant since these proteins are known molecules recruited to the activated TNFRs, acting as scaffolds in the building of the signalosome (*37*). TRAF1, TRAF2, and TRAF3 were found associated with CD137, thus confirming previous results indicating physical and/or functional associations of these proteins with CD137 (*28*–*30*, *34*, *47*, *48*). We also found evidence of the association of TRAF5 to CD137. A similar result was obtained when CD137 stably transfected Jurkat T cells were used to assess the components of CD137 signaling complex. In this case, TRAF2, TRAF3, and TRAF5 were found in the CD137 immunoprecipitates (Fig. 1B and data S2). TRAF1 was absent in the case of the Jurkat transfectants because it is not expressed in such transformed T cells.

cIAP1 was also found associated with CD137 in both activated primary human CD8 T cells and Jurkat transfectants, while cIAP2 was only found in CD137 immunoprecipitates from primary human CD8 T cells (Fig. 1, A and B). However, Western blot analyses showed that both cIAP1 and cIAP2, as well as TRAF2, were detected in CD137 immunoprecipitates from the CD137-transfected Jurkat T cells (Fig. 1, C and D). Since Jurkat express low levels of cIAP2 mRNA (fig. S1D), it is not surprising that is not detected in the coimmunoprecipitates. cIAP2 appears to be under a very dynamic transcription regulation as compared to cIAP1 (*49*).

As shown in Fig. 1E, tryptic peptides common for both cIAP1 and cIAP2 and exclusive of each of the cIAP proteins were identified in the coimmunoprecipitates with excellent levels of confidence. Examples of the sequences and spectra of conclusively defining unique peptides for cIAP1 and cIAP2 are presented in Fig. 1, F and G, respectively.

To further offer proof for a CD137 physical association with cIAPs, 293T cells that had been transiently transfected with a hCD137-GFP (green fluorescent protein) construct were intracellularly stained with an anti–pan-cIAP mAb and stimulated with the CD137 agonist mAb 6B4 for 20 min. As can be seen in Fig. 1 (H and I), there was an important level of CD137 colocalization with cIAPs, more evident upon internalization of CD137 following a 20 min culture.

HOIL-1 and HOIP were also detected in the CD137 immunoprecipitates, thus providing the first physical evidence of the participation of the LUBAC complex in CD137 signalosomes. The LUBAC complex might associate to CD137 signalosome through its reported interactions with TRAF2 (*50*) and cIAP1 (*51*). Furthermore, the A20 (TNFAIP3) protein was found in the CD137 immunoprecipitates from human CD8 and Jurkat T cells, thus confirming previous results from our laboratory (*52*).

The E3 ubiquitin ligase ITCH was also identified with high confidence in CD137 immunoprecipitates. ITCH is a E3 ubiquitin ligase that has been shown to be part of the A20 and CYLD ubiquitin-editing complexes, aiming at the regulation/termination of NF-κB signaling (*53*).

The IKK complex proteins IKKβ and NEMO appeared to be physically associated with the CD137 signaling complex. These proteins were found in the CD137 immunoprecipitates from primary CD8 T cells. It has been shown that TRAF2 phosphorylation and the subsequent K63 self-ubiquitination of TRAF2 allow the recruitment of the IKK complex to activated TNFR1 (*54*).

We have also detected in the CD137 immunoprecipitates galectin-8, which is a member of the galectin family of soluble β-galactoside–binding proteins. Thus, galectin-8 seems to also participate in the extracellular regulation of CD137 clustering as it has been described for galectin-9 (*55*) and galectin-3 (*56*). An exclusive peptide of galectin-3 was also identified in the immunoprecipitates (data S1).

Using databases of protein interactomes, we modeled the protein-to-protein interactions that may be ensembled in the 4-1BB (CD137) signaling complex according to published literature (fig. S1B). Ingenuity Pathway Analysis (IPA) confirmed that many proteins in the coprecipitates are related to TNFR family signaling pathways including that of CD137 (4-1BB) (fig. S1C).

### cIAPs are required for CD137 signaling to activate NF-κB and MAPKs

The unequivocal presence of cIAPs in the CD137 signalosome prompted us to ascertain the actual involvement of cIAPs in CD137 function. SMCs target cIAP1 and cIAP2 causing their rapid degradation (*46*) and as drugs are currently undergoing clinical trials for malignant indications (*57*, *58*). To explore the extent of cIAP involvement in the signaling pathway and function of CD137, we used BV6, birinapant, and xevinapant as three highly active SMCs (*59*, *60*) and we also used transfections of DN mutants of cIAP1 (H588A),cIAP2 (H574A), and TRAF2 ΔRING (a truncated form of TRAF2 that lacks the RING domain) (*38*).

**Fig. 2.**
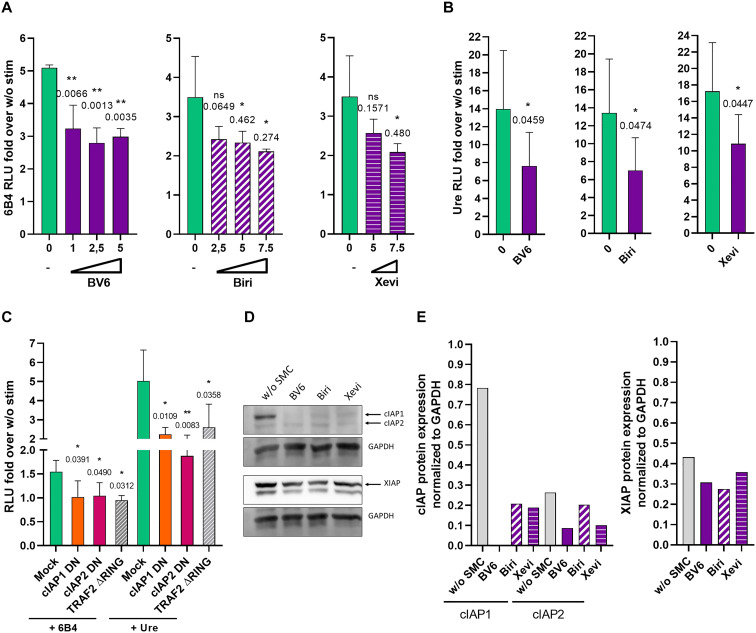
cIAP1 and cIAP2 mediate NF-κB activation via CD137 signaling. (**A**) Jurkat cells stably transfected to express CD137 and an NF-κB luciferase reporter system were stimulated for 6 hours with agonist anti-CD137 (6B4) and studied for luciferase activity upon cotreatment in culture with the SMC compounds BV6, birinapant (Biri), and xevinapant (Xevi) as indicated at different concentrations. RLU, relative light unit. (**B**) Similar experiments as in (A) but using urelumab to stimulate the reporter Jurkat cell cultures and using fold change of luciferase activity as a readout. (**C**) Experiments as in (A) but using Jurkat subcultures that had been mock-transfected or electroporated with expression cassettes encoding cIAP1 and cIAP2 DN variants with nullifying point mutations in the RING domain or TRAF2 ΔRING, 24 hours before the experiments. Experiments were independently repeated three times rendering comparable results, and mean ± SD are provided. One-way analysis of variance (ANOVA) Dunnett’s multiple comparisons and Student’s *t* tests were used as needed for statistical comparisons, and *P* values are shown in comparison to stimulation without SMCs. (**D**) Western blot evidence for cIAP degradation in Jurkat cells by the SMCs, while XIAP is preserved. GAPDH was stained as a sample loading control. (**E**) Densitometries corresponding to (D).

First, we assessed the effect of the cIAP degraders in CD137-mediated NF-κB signaling. For these experiments, CD137 stably transfected Jurkat cells also carrying a luciferase reporter gene system under the control of an NF-κB promoter (*20*) were stimulated with agonist anti-CD137 mAb (6B4). As shown in Fig. 2A, treatment with either BV6 (left), birinapant (middle), and xevinapant (right) clearly inhibited NF-κB activation after 6-hour treatments while minimally affecting cell viability in these cell culture conditions. Furthermore, similar experiments with the clinical grade anti-CD137 mAb urelumab (*14*) rendered similar results upon treatment with the SMCs (Fig. 2B).

A comparable result was obtained when CD137 stably transfected Jurkat cells were transfected with cIAP1 and cIAP2 DN variants that have a nullifying point mutation in their RING E3 ubiquitin ligase catalytic domains. The transfection of either cIAP DNs also caused a considerable reduction in NF-κB activation (Fig. 2C). This means that the catalytic activity of cIAPs is also somehow required. Transfection of TRAF2 ΔRING also resulted in a reduction of NF-κB activation. The specificity of the SMCs for cIAPs was checked by Western blot that showed degradation of cIAPs, while XIAP presence in the lysates was preserved.

**Fig. 3.**
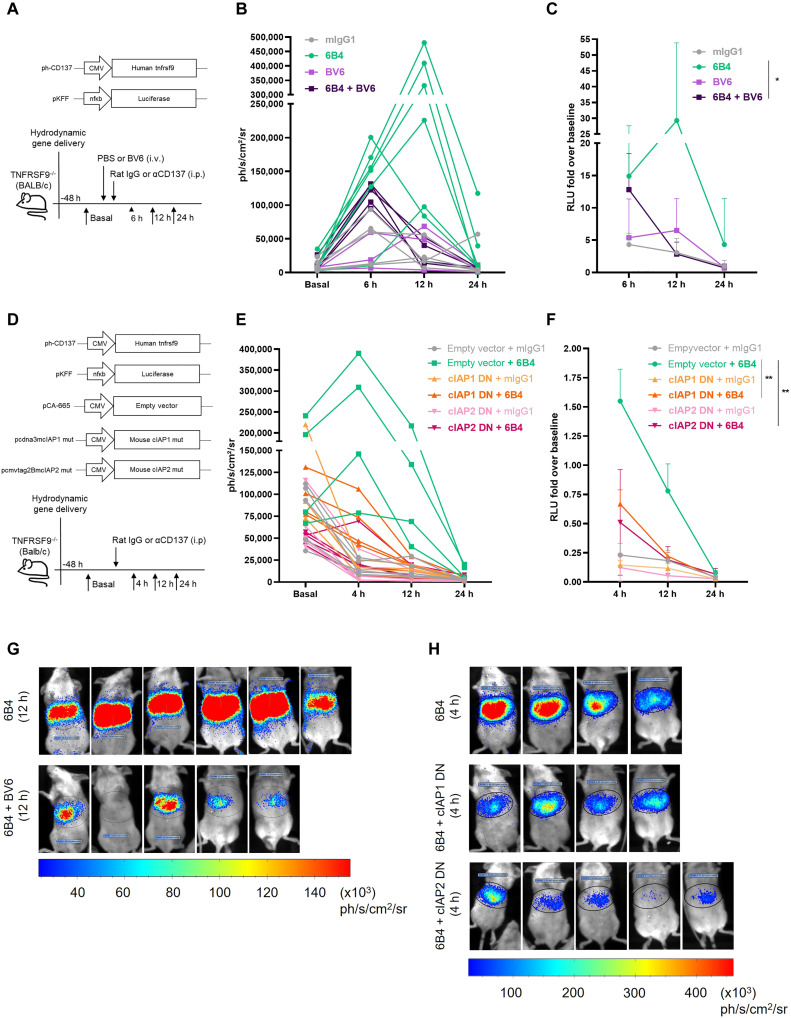
In vivo cIAP involvement in the NF-κB activation induced by anti-CD137 agonist mAbs. (**A**) Schematic representation of experiments in which CD137^−/−^ mice (to completely avoid interference with endogenous mouse CD137) were hydrodynamically gene-transferred to the liver with expression cassettes encoding human CD137 and a luciferase reporter system controlled by NF-κB consensus sequence. Mice were intraperitoneally (i.p.) treated with agonist anti-CD137 mAb (6B4), control antibody, or intravenous (i.v.) BV6 as indicated. (**B**) Light emission from the upper abdominal region of the individual mice treated as color-coded in the legend. (**C**) Representation of data in (B) as fold change relative to the baseline light emission. (**D**) Schematic representation of experiments as in (A) but cotransferring cIAP1 or cIAP2 gene expression cassettes encoding for the cIAP DN mutants. (**E**) Representation of the color-coded results from individual mice, corresponding to the different gene transfer and treatment groups. (**F**) Results as in (E) but showing data in terms of fold change over baseline light emission. (**G** and **H**) Representative bioluminescence images from experiments as those in (B) and (E), respectively. Two-way ANOVA tests were used for statistical comparisons.

To study the signal inhibition effect in vivo, we used a hydrodynamic gene transfer approach to mouse hepatocytes. In this experimental system, about 10 to 15% of hepatocytes (*61*) get cotransfected with human CD137-encoding cDNA and the NF-κB reporter luciferase gene constructs (*38*). In this system, upon systemic injection of luciferin, emission of light from the upper abdominal area reflects NF-κB transcriptional activity (*38*). In this experimental setting (Fig. 3A), we found that injection of the agonist 6B4 anti-CD137 mAb results in prominent light emission, which is strongly reduced if the SMC BV6 is intravenously infused to the mice (Fig. 3, B and C). In a similar system (Fig. 3D), we also tested the cotransference of cIAP1-DN or cIAP2-DN gene expression cassettes or an empty plasmid to the liver. Again, the coexpression of a DN variant of cIAP1 or cIAP2 resulted in a clear reduction of NF-κB activity (Fig. 3, E and F). Representative bioluminescence images are shown in Fig. 3 (G and H) for BV6 and cIAP-DN approaches, respectively.

Experiments were also performed to learn the effect of cIAP degradation with SMCs on the MAPK pathways toward the CD137 costimulation signals. Western blot analyses showed that in primary preactivated CD8 T cells, 4-1BB stimulation with plastic-bound CD137L led to up-regulation of phosphorylated p38. BV6 treatment reduced these effects as shown in Western blots (fig. S2A), whose corresponding densitometry is shown (fig. S2B). In starved Jurkat stable CD137 transfectants, we similarly studied phosphorylation of extracellular signal–regulated kinase 1/2 (ERK1/2) showing that degradation of cIAPs attenuated this CD137-elicited signaling events. In all these experiments, degradation of cIAPs was readily induced by BV6 addition to the cultures (Fig. 3, A and C). Such results collectively indicate that cIAP inhibition blocks early events in CD137 signaling and, together, provide evidence for a role of cIAPs in the signal transduction that can be elicited by infusion of agonist anti-human CD137 mAbs such as those used for cancer immunotherapy purposes.

### cIAPs mediate CD137 costimulation of mouse and human CD8 cells

**Fig. 4.**
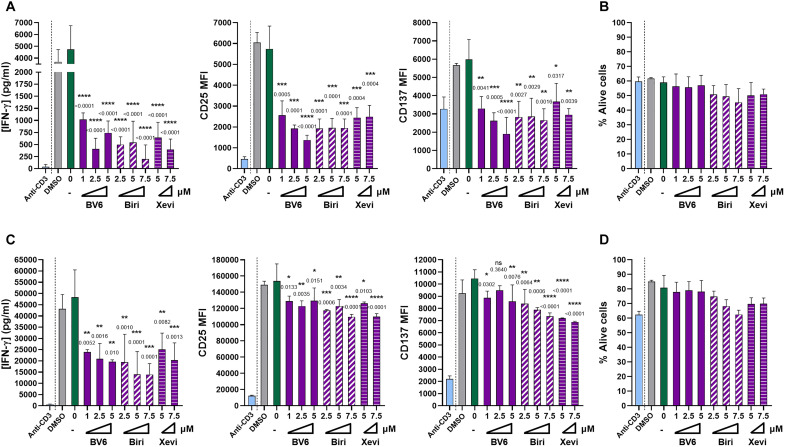
CD137 costimulation of human and mouse CD8 T cells is dependent on cIAPs. (**A**) Experiments on 48-hour cultures of CD8 isolated human T lymphocytes stimulated with microbeads coated with anti-CD3 and anti-CD137 (6B4) in the presence of the indicated concentrations of the SMCs BV6, birinapant, or xevinapant. The concentration of IFN-γ released to the tissue culture supernatants and the levels of surface expression of CD25 and CD137 were assessed. MFI, mean fluorescence intensity (arbitrary units). (**B**) Viability in the cell cultures assessed with Zombie NIR staining. (**C**) Magnetically isolated mouse CD8^+^ splenocytes were cultured on plates coated by anti-CD3 and anti-CD137 (3H3) mAbs. Forty-eight hours later, cells and tissue culture supernatants were collected. When indicated, the SMC compounds were added at the indicated concentrations. The concentration of IFN-γ in the tissue culture and surface expression of CD137 and CD25 at the end of the 48-hour cultures are provided. (**D**) Cell viability in experiments as in (C) as assessed with Zombie NIR using flow cytometry. Experiments were repeated three times in both human and mouse experiments, and means ± SD are shown. CD8^+^ T cells from three independent healthy donors were used. Statistical comparisons with the cultures stimulated without SMCs are provided (one-way ANOVA Dunnett’s multiple comparisons).

CD8 T cells can be stimulated in culture with anti-CD3 + anti-CD137 mAb coating the surface of the tissue culture plates or with microbeads coated with anti-CD3 + anti-CD137. Isolated peripheral blood human CD8 cells were cultured with anti-CD3 + anti-CD137–coated microbeads adding in some instances one of the three SMCs (BV6, birinapant, or xevinapant). Figure 4A shows that SMCs reduced the release of interferon-γ (IFN-γ) to the supernatant and the induction of surface CD25 and CD137 expression (Fig. 4A), while cell viability was preserved (Fig. 4B). Following similar protocols with CD8^+^ T mouse splenocytes stimulated with anti-CD3 + anti-CD137 bound to the plastic culture plates, the effects of the three SMCs were recapitulated (Fig. 4, C and D), indicating that cIAP involvement in CD137 signaling is conserved between primates and rodents.

Figure S4B shows confocal microscopy evidence that BV6 addition did not result in any down-regulation of CD137 internalization in human preactivated CD8 T cells upon CD137 ligation by Alexa Fluor 488 (AF488)–conjugated anti-CD137 mAb. Therefore, cIAP function and CD137 internalization are seemingly uncoupled phenomena.

### cIAPs are required for the immunotherapeutic antitumor effects of anti-CD137 agonist mAbs

**Fig. 5.**
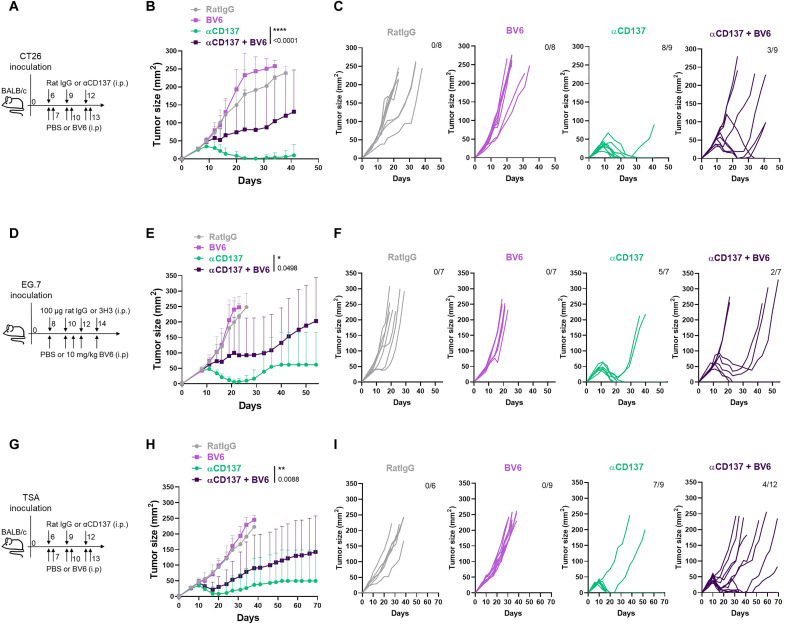
Immunotherapy with agonist αCD137 mAbs is dependent on cIAPs. (**A**) Scheme of experiments in which mice bearing CT26-derived established tumors were treated as indicated with anti-CD137 mAb (3H3) or control irrelevant IgG antibody via intraperitoneal injections. When indicated, some of the groups received intraperitoneally the BV6 SMC. (**B**) Mean ± SD and statistical comparisons. (**C**) Tumor size individual follow-up in the indicated groups of mice. The fraction indicates the relative number of mice that achieved complete rejections. These experiments have been repeated twice with similar results. (**D**) Similar experiment as in (A), using C57BL/6 mice subcutaneously engrafted with EG.7 cells. (**E**) Average tumor size follow-up and statistical comparisons. (**F**) Individual size follow-up indicating the fraction of mice completely rejecting their tumors. (**G**) Similar experiment as in (A) by using implanted TSA tumors engrafted in BALB/c mice. (**H**) Follow-up of average tumor sizes and statistical analyses. (**I**) Individual follow-up of the tumor sizes indicating the fraction of mice completely rejecting their tumors. Two-tailed ANOVA was used for statistical analyses.

CT26-derived tumors implanted in syngeneic BALB/c mice are highly susceptible to treatment with anti-CD137 mAb (*62*), achieving complete rejections in most cases. To determine whether treatment with the BV6 SMC would have any effect on CD137-mediated antitumor activity, we performed the experiments schematized in Fig. 5A. Results are shown in Fig. 5 (B and C).

As expected, the treatment of CT26-engrafted mice with anti-CD137 mAb resulted in very efficient tumor regression in eight of nine mice and a substantial delay in tumor progression in the other treated mouse. In contrast, the treatment of mice with the BV6 SMAC inhibitor caused a striking reduction of CD137-elicited antitumor effects. BV6 treatment resulted in unaltered tumor progression (six of nine mice) or in a delay in tumor regression (three of nine mice) (Fig. 5, B and C). Of note, BV6 SMC treatment alone had no effect on tumor growth as compared to mice treated with an irrelevant IgG, thus indicating that BV6 lacks any antitumor effect on CT26-derived tumors.

A similar experimental approach was undertaken with tumors derived from the EG.7 lymphoma (Fig. 5, D to F) and the TS/A breast cancer (Fig. 5, G to I) cell lines. Again, BV6 treatment hindered the antitumor efficacy of anti-CD137 mAb, while BV6 had no effect by itself on tumor progression.

Hence, these observations strongly indicate that hampering CD137-cIAP signaling in T cells, as a result of inducing systemic degradation of cIAPs with an SMC, abrogates the ability of anti-CD137 mAb to mediate antitumor immunotherapeutic effects.

In keeping with these results, examination of the cellular composition of the CT26 tumor microenvironment showed that anti-CD137 treatment caused an increase in CD8 T cell infiltration that was markedly reduced by BV6 treatment (fig. S4, A and B). At the time of tumor excision, some CD137-mediated therapeutic effects were evident and early tumor shrinkage was not seen upon cotreatment with BV6 (fig. S4C). Of note, BV6 treatment also reduced NK cell infiltrates and increased the presence of F4/80^+^ macrophages, while the numbers of CD11b^+^ and Ly6G^high^ myeloid cells remained unchanged (fig. S4D). When studying tumor tissue sections using multiplex tissue immunofluorescence, similar effects were substantiated showing CD137-mediated increases of CD3, CD8, and CD4 T cells that were reduced upon BV6 cotreatment (fig. S4, E and F). This was also evident for activated CD8^+^ Ki67^+^ T lymphocytes (fig. S4F). Of note, CD11b cells in the infiltrates were only marginally reduced by BV6, while regulatory T cells (T_regs_) (CD4^+^ FOXP3^+^) were markedly reduced (fig. S4, E and F). Overall, these effects in the tumor tissue microenvironment are consistent with the loss of therapeutic efficacy of CD137 mAb upon cotreatment with SMCs.

### cIAPs are involved in the signaling from CD137-based CARs

The cytoplasmic tails of CARs to be efficacious require costimulatory domains (*26*). The intracytoplasmic sequence of CD137 that is used for these purposes is cloned in tandem along with CD3ζ to achieve so (*26*).

**Fig. 6.**
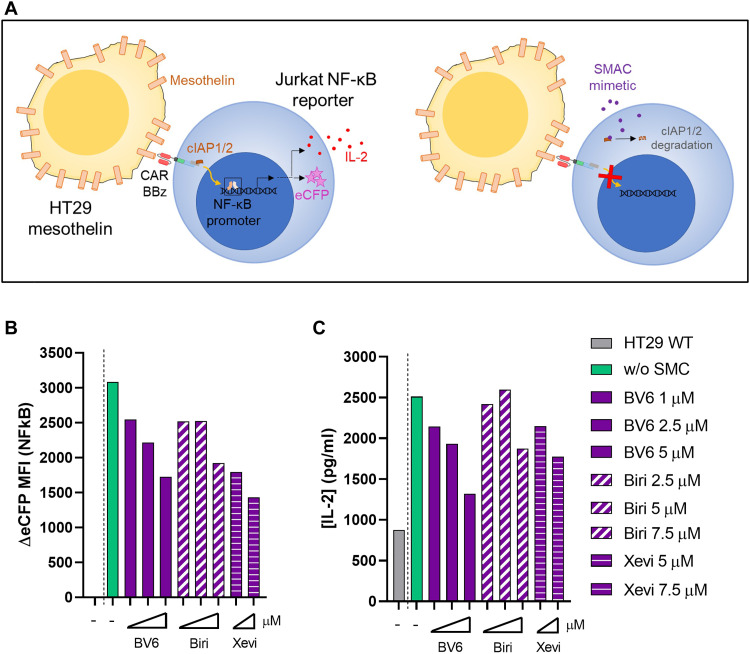
cIAPs are involved in signaling from CD137-encompassing CARs. (**A**) Schematic representation of experiments in which Jurkat cells reporting NF-κB activity expressing eCFP were lentivirally transduced with an anti-mesothelin BBz CAR and cocultured for 12 hours with mesothelin stable transfectants in HT29. Cocultures were performed in the presence or absence of SMCs. (**B**) Increased expression of eCFP controlled by NF-κB promoter determined by flow cytometry upon culture with different concentrations of the SMCs as indicated. (**C**) Measurements of IL-2 concentrations in the corresponding tissue culture supernatants. Three independent experiments in triplicates were performed rendering comparable results.

To study if cIAPs were involved in the function of CARs encompassing 4-1BB, we used a BBz CAR recognizing mesothelin that was transduced into reporter Jurkat cells in which the fluorescent protein eCFP is controlled by an NF-κB–responsive promoter. CARs in Jurkat cells were stimulated during 12-hour cocultures with HT29 human colon cancer cells stably transfected to express mesothelin on their surface (see the scheme of the experimental system in Fig. 6A).

As can be seen in Fig. 6B, cultures in the presence of BV6, birinapant, or xevinapant reduced the increase in NF-κB signaling due to the coculture with mesothelin^+^ HT29 cells in a concentration-dependent manner. Moreover, in supernatants collected from similar cocultures, the three SMCs resulted in a reduction of the amount of IL-2 released to the supernatant. These experiments provide strong evidence for a role of cIAPs in mediating signaling events from CD137-encompassing CARs, such as these approved for clinical use.

## DISCUSSION

Signaling by several members of the TNFR family can potently costimulate T lymphocytes (*63*, *64*). These include CD137 (4-1BB), OX40 (CD134) (*65*), CD27 (*66*), GITR (*67*), or the artificial expression of LTβR in T cells (*68*). The pattern of surface expression of such receptors is different and depends on the activation status, the T lymphocyte subsets, and their differentiation toward memory and other factors (*69*). Signaling via such receptors is under active investigation due to its translational potential to modulate immune responses (*19*, *21*) and their potential as targets for T cell costimulation in immunotherapy (*63*, *64*). Bereft of any known enzymatic activity, these TNFR members rely on interactions with binding partners to relay biochemical signals that elicit NF-κB and MAPK activation (*70*). The outcome of the response will also depend on the levels of expression of the distinct proteins that might participate in the signalosomes of TNFR family members in each particular T cell subset.

As a key example, understanding CD137 (4-1BB) signaling is of special interest because CD137 is a pursued target in immunotherapy. Experimental agonistic anti-CD137 mAbs are in the clinics (*71*), and new CD137-targeting immunotherapeutic agents, including mono-, bi-, and multi-specific antibodies, are being developed (*11*, *72*). Furthermore, CAR cassettes often rely on the cytosolic tail of CD137 to transmit costimulatory signals into the transduced T and NK cells (*26*, *73*).

When considering CD8 T cell activation, CD137 becomes expressed upon antigen recognition by the T cell receptor (TCR) and is favored by CD28 costimulation (*74*). Once ligated, CD137 is reportedly able to signal through TRAF2 via a number of K63 polyubiquitination reactions (*38*). However, the precise nature of the enzymes and substrates participating in CD137 signaling is incompletely understood at present.

Here, we provide coimmunoprecipitation and mass spectrometry evidence to identify components of the CD137 signalosome. We show that several TRAF family members (TRAF2, TRAF1, TRAF3, and TRAF5) as well as cIAP1 and cIAP2, proteins of the LUBAC complex (HOIL-1 and HOIP), A20, and members of the IKK complex are components physically associated with the CD137 signalosome. Most of these proteins have been already implicated in the signaling complex of other members of the TNFR family (*40*) and were expected to participate in CD137 signaling (*33*, *37*), but here, we report coimmunoprecipitation evidence of their physical presence in the CD137 signaling complex.

We have also identified some proteins not previously implicated in CD137 signaling. This is the case of TRAF5. This member of the TRAF family is less characterized than other TRAFs. It is most homologous to TRAF3 but functionally similar to TRAF2 (*75*). Its presence implies that additional TRAF trimer compositions might interact with the activated CD137, adding yet another level of complexity to CD137 signaling (*37*). The presence of ITCH, an A20 binding protein and a regulator of its activity, highlights the role of A20 to modulate CD137 signaling (*52*).

The binding of galectin-8 to CD137 was not previously reported but galectin-9 reportedly associates to the extracellular region of CD137, positively regulating CD137 activities by facilitating CD137 aggregation (*55*). However, galectin-9 was not observed in our coprecipitates probably as a result of our experimental conditions. Galectin-3, which is identified in the coimmunoprecipitates, has been recently reported to interact with CD137 and reduce its activity by crosslinking soluble and membrane-bound 4-1BB (*56*). These results further support a potentially important roles of galectins (*76*) in regulating CD137 bioactivities.

We have not found evidence of CD137 association to transforming growth factor β–activated kinase (TAK) and TAK binding proteins TAB1, TAB2, and TAB3 that reportedly interact with K63-polyubiquitinated TRAF2 (*77*).

Of special interest for us was the demonstration of the physical association of cIAPs with CD137. An involvement of cIAPs in CD137 signaling was expected due to their role in the regulation of NF-κB2, which is induced upon CD137 activation (*33*). Besides, the intimal structural and functional relation between TRAF2 and cIAP1 and cIAP2 strongly supports that these proteins often work in tandem. The crystallization and analysis of the TRAF:cIAP complexes (*43*) showed that a single cIAP1 or cIAP2 tightly binds to the coiled coil of TRAF2 homotrimers and TRAF1:(TRAF2)_2_ heterotrimers through their BIR1 domain. Our colocalization confocal microscopy results argue in the same direction.

A working model for CD137 signaling is that TRAF2-cIAPs complexes jointly signal via K63 polyubiquitination of substrates that include TRAF2 and NEMO to act as docking sites for subsequent signaling components. A20 is probably associated with the complex to regulate and fine-tune signaling as a K63 deubiquitinase (*52*). IKK complex members are found in the CD137 signalosome most likely as downstream polyubiquitination substrates. The presence of LUBAC complex components is also regulated by K63 ubiquitination (*70*) and is also likely acting downstream of TRAF2-cIAPs. In this line, our coprecipitation findings suggest a role for linear ubiquitination in CD137 signaling that we are currently investigating. To ascertain, how all this is molecularly accomplished requires further experimental studies, but our results with DN variants argue that the E3 ubiquitin ligase activity of cIAPs is required. The functional interaction of CD137 and cIAPs was previously suggested using a cIAP-DN transgenic mouse strain (*44*).

Here, we have substantiated the critical contribution of cIAPs in CD137 signaling by various experimental approaches. First, we show using SMCs and cIAP1 and cIAP2 mutants lacking E3 ubiquitin ligase activity that fully active cIAPs are required for efficient CD137-elicited NF-κB activity. Pharmacological evidence was substantiated with three different SMCs that readily induced the degradation of cIAPs but, importantly, had no effect on XIAP integrity.

We further confirmed in vivo this result using treatment conditions similar to CD137-based immunotherapy. In this setting, we engineered reporter mice in the liver by hydrodynamic injection of plasmids expressing human CD137 and NF-κB–controlled luciferase. Pharmacological and genetic evidence in that model conclusively demonstrated the involvement of cIAPs in CD137 in vivo signaling.

Furthermore, performing experiments in a well-stablished in vivo cancer immunotherapy model responding to agonist anti-CD137 mAb treatment, the BV6 SMC hampered the antitumor efficacy of CD137 agonists. These results in three transplantable mouse tumor models prove that cIAPs are necessary for the therapeutic effects elicited via CD137 stimulation with agonist mAbs. The observation in the changes in the leukocyte composition of the CT26 tumor microenvironment upon treatment is consistent with these findings.

The observation of a deleterious role of SMCs in cancer treatment might be counterintuitive, since these compounds have been developed as antitumor agents to promote apoptosis in tumor cells. However, while SMCs are promising drugs, it should be taken into consideration that SMCs might also have a deleterious effect on the patient’s antitumor immune response. Caution should be specially exercised when CD137-based immunotherapies are considered, including antibody-based CD137-targeting immunotherapeutic tools (*11*, *72*) and also the use of CD137-based CAR constructs (*78*), which rely on the CD137 cytosolic tail to costimulate activity and favor persistence of adoptively transferred cells (*73*). In both instances, they will require unaltered cIAP1 and cIAP2 activity. We provide evidence for a role of cIAPs in signaling events of CD137-encompassing CARs such as those that are U.S. Food and Drug Administration–approved for clinical use (*26*, *27*). Our results showing that SMAC inhibitors reduce NF-κB–mediated cytokine release in activated lymphocytes suggest that transient use of SMCs could mitigate side effects such as cytokine release syndromes.

All things considered, our work provides evidence for CD137 physical and functional association with cIAPs that might extend to other costimulatory members of the TNFR family known also to interact with TRAF2 (*63*). Our findings on a key role of cIAPs for the antitumor effects of CD137 agonists have important translational implications in cancer immunotherapy based on CAR T cells and CD137 agonists that are being developed in the clinic in different combinations (*64*, *72*).

## MATERIALS AND METHODS

### Mice

C57BL/6 and BALB/c mice were purchased from Envigo RMS Spain (Barcelona, Spain). C.Cg-Tnfrsf9^tm1Byk^ (CD137KO BALB/c) mice have been previously described (*79*). Female mice were used at 6 to 9 weeks of age and maintained under specific pathogen–free conditions. All experiments involving animals were approved by the Ethics Committee of Animal Experimentation (CEEA) at the University of Navarra (R-053-22GN).

### Cell lines

CT26 mouse colon carcinoma, EG.7 lymphoma, and TS/A breast cancer cell lines were gifted by M. Colombo (IRCCS Istituto Nazionale dei Tumori, Milano, Lombardia, Italia), K. E. Hellstrom (UW Medicine, Seattle, USA), and L. Galluzzi (Weill Cornell Medical College, New York, New York, USA), respectively. HEK293T cells and HT29 human colon carcinoma cell line were purchased from the American Type Culture Collection (ATCC). HT29 cell line was stably transfected with a sleeping beauty–based plasmid expressing membrane-bound mesothelin using Lipofectamine 2000 (Thermo Fisher Scientific, San Jose, CA, USA). CD137 (4-1BB)–stable transfected Jurkat cell line was purchased from Promega (4-1BB Bioassay, JA2355). eCFP/NF-κB reporter Jurkat cell line was gifted by P. Steinberger (Medical University of Vienna, Vienna, Austria). Cells were grown in RPMI 1640 medium + GlutaMAX (Gibco) supplemented with 10% heat-inactivated fetal bovine serum (FBS), penicillin (100 U/ml), and streptomycin (100 μg/ml) at 37°C with 5% CO_2_. 2-Mercaptoethanol (50 μM) was also added in case of mouse-derived cells.

### Isolation of primary mouse and human T cells

Peripheral blood mononuclear cells (PBMCs) were density gradient–separated (Ficoll-Paque PLUS, GE Healthcare) from blood of healthy donors (*n* = 3). The remaining erythrocytes were lysed by ACK buffer. CD8 T cells were freshly isolated by a negative magnetic selection kit (human CD8 T cell isolation kit, Miltenyi Biotec) according to the manufacturer’s instructions.

Spleens from naïve C57BL/6 mice were mechanically processed and ACK lysed. Mouse CD8 T cells were obtained from splenocytes by a negative mouse CD8 T cell isolation kit (Miltenyi Biotec).

### Microbead coating and preparation

Anti-CD3ɛ (OKT3), anti-CD137 (6B4), and mIgG1 isotype control (MOPC-21) mAbs were covalently coupled to Dynabeads M-450 and M-280 Tosylactivated (Thermo Fisher Scientific) according to the manufacturer’s instructions.

### CD8 T cell activation

For immunoprecipitation experiments, isolated human CD8 T lymphocytes were preactivated with Dynabeads Human T-Activator CD3/CD28 (Thermo Fisher Scientific) at an E:T (Effector:Target) ratio 1:1.5 in complete RPMI medium plus IL-2 (50 U/ml) to prime and induce 4-1BB expression.

For activation experiments, human and mouse CD8 T cells were activated using anti-CD3 (OKT3 or 17A2) and anti-CD137 (6B4 or 3H3) mAb coupled to microbeads or plate-coated for 48 and 72 hours, respectively. BV6 (Selleckchem), birinapant (Selleckchem), and xevinapant (Selleckchem) were added together with the agonist antibodies at the concentrations described.

### Immunoprecipitation and Western blot analyses

CD137 (4-1BB)–stable transfected Jurkat cells and preactivated human CD8 T cells (see above) were incubated with MG132 (5 μM) to avoid proteasomal degradation. Next, cells were stimulated in CD137 (4-1BBL)–Fc–coated (AcroBiosystem) plates for 15 min and lysed with a Brij96-based buffer [20 mM tris-HCl (pH 7.5), 150 mM NaCl, 1 mM EDTA, 1% Brij96] complemented with cOmplete EDTA-free Protease Inhibitor Cocktail (Roche) and PhosSTOP (Roche). Lysates were centrifuged (18,000*g*, 5 min) and then incubated with αCD137 (6B4)– or control isotype mIgG1–coated Dynabeads m280. Immunocomplex was eluted in Laemmli Buffer 1× (without β-mercaptoethanol) at 95°C, 5 min and stored at −20°C.

Immunoprecipitates were analyzed by SDS–polyacrylamide gel electrophoresis (PAGE)/immunoblotting using specific antibodies against CD137 (5D1, own hybridoma), TRAF2 (F2, Santa Cruz Biotechnology), cIAP1 (D5G9, Cell Signaling Technology), and cIAP2 (E40, Abcam). To avoid interference with the mAb used in the pull-down (6B4), anti-CD137 and anti-TRAF2 mAbs were biotinylated using the EZ-Link Sulfo-NHS-LC-Biotinylation Kit (Thermo Fisher Scientific) according to the manufacturer’s instructions.

### S-TRAP digestion

Protein digestion was performed directly in the S-Trap filter (Protifi, Huntington, NY, USA) following the manufacturer’s procedure with slight modifications (*80*). The samples were reduced and alkylated with TCEP (Tris Carboxy Ethyl Phosphene) and CAA (Chloroacetamide) and digested by trypsin. Each digestion result was washed using StageTip C18 before liquid chromatography electrospray ionization tandem mass spectrometric (LC-ESI-MS/MS) analyses.

### Liquid chromatography and mass spectrometry analysis

An aliquot of each fraction (500 ng) was subjected to 1D-nano LC ESI-MS/MS analysis using an Ultimate 3000 nano HPLC system (Thermo Fisher Scientific) coupled online to an Orbitrap Exploris 240 mass spectrometer (Thermo Fisher Scientific). Peptides were separated in a 50 cm × 75 μm Easy-spray PepMap C18 analytical column at 45°C at a flow rate of 300 nl/min using a 120 min gradient ranging from 2% to 95% mobile phase B [mobile phase A: 0.1% formic acid (FA); mobile phase B: 80% acetonitrile (ACN) in 0.1% FA]. The loading solvent was 2% ACN in 0.1% FA, and injection volume was 5 μl.

Data acquisition was performed using a data-dependent top-20 method, in full-scan positive mode, scanning 375 to 1200 mass/charge ratio (*m*/*z*). Survey scans were acquired at a resolution of 60,000 at *m*/*z* 200, with normalized automatic gain control (AGC) target adjusted to 300 (%) and a maximum injection time (IT) selected to AUTO. The top 20 most intense ions (charges ranging from 2 to 5) from each MS1 scan were selected and fragmented via higher-energy collisional dissociation (HCD). Resolution for MS2 spectra was set to 45,000 at *m*/*z* 200, with AGC target of 100 and a maximum ion IT in AUTO. Isolation of precursors was performed with a 0.7 *m*/*z* window, dynamic exclusion was set to 45 s, and the HCD collision energy was set to 32.

### Proteomics data analysis and sequence search

Raw instrument files were processed using Proteome Discoverer (PD) version 2.4 (Thermo Fisher Scientific). MS2 spectra were searched combining four search engines [Mascot (v2.7.0), MsAmanda (v2.4.0), MsFragger (v3.1.1), and Sequest HT] and a target/decoy database containing *Homo sapiens* protein sequences downloaded from UniProt Knowledgebase Database. Static modifications with carbamidomethylation on cysteine were considered, while oxidation of methionine residues (+15.9949 Da) was set as a dynamic modification. A maximum number of two tryptic miscleavages was allowed. Peptide precursor and MS/MS mass tolerance were set to 10 ppm and 0.02 Da, respectively. The false discovery rate (FDR) for proteins, peptides, and peptide spectral match (PSM) peptides was set to 1%. The quantification values for proteins were calculated using the summed abundance of all peptides considered for the identification.

### Plasmids

Human CD137-GFP plasmid (pCMV6-CD137-GFP) and TRAF2 ΔRING plasmid were previously used (*38*). pcdna3.1 hciap1mut (Addgene plasmid #8337; http://n2t.net/addgene:8337; RRID:Addgene_8337), pcdna3.1 hciap2mut (Addgene plasmid #8339; http://n2t.net/addgene:8339; RRID:Addgene_8339), pcdna3mciap1 (Addgene plasmid #11476; http://n2t.net/addgene:11476; RRID:Addgene_11476), and pcmvtag2Bmciap2 mut (Addgene plasmid #11464; http://n2t.net/addgene:11464; RRID:Addgene_11464) were gifts from J. Ashwell. A mesothelin expression plasmid based on pT4-HB was designed based on a sleeping beauty tool to favor insertion available in our laboratory.

### CD137:cIAP colocalization confocal microscopy

HEK293T cells were transiently transfected with hCD137-GFP plasmid using Lipofectamine 2000, and CD137 surface expression was confirmed 24 hours by fluorescence microscopy. After 20 min stimulation with anti-CD137 6B4 mAb, 293T transfectants were fixed with 4% paraformaldehyde (PFA) and permeabilized with phosphate-buffered saline (PBS) + 1% Triton X-100 (Sigma). Subsequently, cells were stained with anti–pan-cIAPs (MAB3400, R&D Systems) mAb at 4°C overnight. Finally, a secondary anti-mIgG2a-AF647 mAb was used to detect anti–pan-cIAP mAb. Images were collected using an LSM 800 confocal microscope (Zeiss). Overlap of both CD137 and cIAP signals was analyzed using IMARIS software.

### cIAP1, cIAP2, TRAF2, and HER2 expression by reverse transcription quantitative PCR

Total RNA was extracted from Jurkat cells and activated human CD8 T cells using a Maxwell RSC simplyRNA tissue kit (Promega) in a Maxwell RSC 48 instrument. M-MLV reverse transcriptase (Invitrogen) and random primers (Invitrogen) were used to obtain cDNA. Quantitative polymerase chain reaction (PCR) was carried out with iQ SYBR Green Supermix (Bio-Rad) in the CFX Connect Real-Time PCR Detection System (Bio-Rad) following the program: 95°C 3 min; [95°C 15 s, 60°C 15 s, 72°C 25 s, 76°C 5 s, 78°C 5 s, 80°C 5 s] x42; 64°C 5 s, 95°C 50 s, 22°C 30 s (SYBR fluorescence was captured in the underlined steps). The primer strategy was based on the following: cIAP1 (forward: 5′- TCGAGGACTAACCCCTACAGT-3′, reverse: 5′-GGCAAAGCAGGCTACCCTAT-3′), cIAP2 (forward: 5′-CAACAGATCTGGCAAAAGCA-3′, reverse: 5′-TTGCTCAATTTTCCACCACA-3′), TRAF2 (forward: 5′-CAAAGCCCCTCTTGGGAGAC-3′, reverse: 5′- CTGCACCTTGCTACTCAGGG-3′), and HER2 (forward: 5′-GCAGGGAAACCTGGAACTCA-3′, reverse: 5′-TCTCCATTGTCTAGCACGGC-3′).

### Electroporation

Jurkat cells expressing CD137 were electroporated with 10 μg of plasmid encoding for DN cIAP variants or a version of TRAF2 lacking RING domain by an exponential protocol (300 V, 950 μF, and ∞Ω) using the Gene Pulser Xcell Electroporation System (Bio-Rad).

### cIAP degradation by SMCs

CD137-expressing Jurkat cells were cultured in the presence or absence of SMCs (BV6, birinapant, and xevinapant) for 1 hour. After incubation, cells were lysed using radioimmunoprecipitation assay (RIPA) buffer for 30 min. Lysates were centrifuged (18,000*g*, 5 min) and stored at −20°C.

Cell lysates were analyzed by Western blot, staining the membranes with specific antibodies against cIAP1 (Cell Signaling Technology) and cIAP2 (Abcam) or XIAP (Cell Signaling Technology). Glyceraldehyde-3-phosphate dehydrogenase (GAPDH) was used as the loading and densitometry control.

### Analyses of MAPK pathways

Purified CD8 T cells were preactivated with Dynabeads Human T-Activator CD3/CD28 (1:1.5) overnight. Cells were rested in clean culture medium for 4 hours and then stimulated with 4-1BBL–Fc–coated plates at the times described in the presence or absence of BV6 (2.5 μM). Protein extraction was performed with RIPA buffer. Lysates were centrifuged (18,000*g*, 5 min) and stored at −20°C. T cell lysates were analyzed by SDS-PAGE/immunoblotting using specific antibodies against phospho-p38 (Cell Signaling Technology) and total p38 (Cell Signaling Technology).

Similarly, CD137 Jurkat transfectants were stimulated with soluble anti-CD137 6B4 mAb in the presence or absence of BV6. Cells were lysed using RIPA buffer, and cell lysates were analyzed by Western blot. Membranes were stained with anti–phospho-ERK1/2 (Cell Signaling Technology) and anti–total ERK1/2 (Cell Signaling Technology) mAbs. In both instances, GAPDH was used as the loading and densitometry control.

### CD137-internalization immunofluorescence staining

CD8 T cells were preactivated with T cell activator beads CD3/CD28. Before CD137 stimulation through CD137 (4-1BBL)–Fc–coated plates, a certain fraction of cells was preincubated with BV6 2 hours at 37°C to ensure cIAP degradation. After 15 min of CD137 stimulation, cells were fixed with 4% PFA and stained with wheat germ agglutinin (WGA) AF647 (Life Technologies), anti-CD137–AF488 (BioLegend), and HOETSCH. Images were collected using an LSM 800 confocal microscope (Zeiss). Images were analyzed using ImageJ, generating manual region of interest (ROI) in the cytoplasm of each CD8 T cell and quantifying the mean fluorescence intensity of CD137 signal in each cell cytoplasm.

### Hydrodynamic gene-transfer CD137 signaling model

Female CD137KO mice were hydrodynamically transfected with plasmids encoding a luciferase reporter gene system under NF-κB promoter and human CD137 (*38*). Anti-CD137 6B4 mAb was administered with or without BV6, and luciferase activity was evaluated at 6, 12, and 24 hours after mAb infusion using a PhotonIMAGER (Biospacelab). Basal point time was assessed 30 min before mAb treatment. Similar hydrodynamic gene transfer experiment was performed cotransferring plasmids encoding DN cIAP variants.

### Mouse tumor models

To evaluate the role of cIAPs in αCD137 agonist mAb therapeutic efficacy, 5 × 10^5^ CT26 and TS/A tumor cells were subcutaneously inoculated into the right flanks of syngeneic mice. When tumors reached 16 to 25 mm^2^, the mice were randomized into four groups. The anti-CD137 agonist mAb (100 μg; clone 3H3, Bio X Cell) was administered intraperitoneally or three times with a 3-day interval. BV6 (Selleckchem) used at 10 mg/kg per dose was administered by intraperitoneal injections in a similar way. Control mice received intraperitoneal injections of rat IgG and PBS.

Similarly, mice bearing EG.7-derived tumors were treated with 100 μg of 3H3 four times every other day. BV6 was intraperitoneally administered in a concomitant treatment regimen.

### Tumor microenvironment immune infiltration analysis

At the indicated time point in fig. S4A, tumors were excised and weighted for further estimation. One-third of the piece was divided for multiplex immunofluorescence staining. The remaining tumor part was disaggregated after incubation with Collagenase D (400 U/ml) (Roche) and DNase (50 μg/ml) (Roche) for 30 min at 37°C. Tumor cell suspensions were filtered through a 70 μm cell strainer (Thermo Fisher Scientific) and resuspended in PBS. Percoll gradient centrifugation was performed to discard cellular debris.

### CAR transduction and stimulation

eCFP/NF-κB reporter Jurkat cells were transduced with lentivirus encoding for an anti-mesothelin BBz CAR, provided by S. Guedán (Hospital Clinic, Barcelona, Spain) and C. June (University of Pennsylvania, Philadelphia, PA, USA) (*81*). After 6 hours, CAR expression was confirmed by flow cytometry. CAR-transduced Jurkat cells were cocultured with mesothelin^+^ HT29 transfectants for 12 hours. Cells were examined using flow cytometry for eCFP levels of expression, and the concentration of IL-2 was determined in tissue culture supernatants.

### Flow cytometry

Human cells were stained with anti-CD8–BV510 (BioLegend) and anti-CD137–Biot (5D1, laboratory’s own hybridoma). Streptavidin-PE (phycoerythrin) was added to detect 5D1-Biot antibody. FMO was performed as negative control. Mouse T cells were stained with anti-CD8–PECy7 (BioLegend), anti-CD25–APC (allophycocyanin) (BioLegend), anti-CD69–BV510 (BioLegend), anti-CD137–PE (BioLegend), and anti-PD1–PerCPCy5.5 (BioLegend). Rat IgG1-APC (BioLegend), Armenian Hamster–BV510 (BioLegend), Syrian Hamster–PE (BioLegend), and Rat IgG2a-PerCPCy5.5 (BioLegend) antibodies were used as isotype-matched negative controls. Zombie NiR (BioLegend) was used to exclude cell death.

Tumor-derived cell suspensions were generated with a cell strainer and stained with a lymphoid panel using anti-CD45–PB (BioLegend), anti-CD3–PerCP-eF710 (eBiosciences), anti-CD8–BUV395 (BD Biosciences), anti-CD4–BUV496 (BD Biosciences), anti-CD11b–PECy7 (BioLegend), anti-CD19–PECy7 (BioLegend), and anti-F4/80–PECy7 (BioLegend). The myeloid immunostaining panel consisted of anti-CD45.2–FITC (BioLegend), anti-CD11b–BUV395 (BD Biosciences), anti-CD11c–PerCPCy5.5 (BioLegend), anti-Ly6G–BV510 (BioLegend), anti-Ly6C–AF647 (BioLegend), anti-F4/80–PECy7 (BioLegend), and anti-CD49b–PE (BD Biosciences). PromoFluor-840 (PromoCell) was used to exclude cell death.

CAR expression in transduced Jurkat cells was confirmed using a secondary anti-human IgG (H+L)-Biot (Jackson ImmunoResearch) followed by a SAV-PE (BioLegend). Samples were acquired on a CytoFlex S or LX system (Beckman Coulter). Analyses were performed using CytExpert software (Beckman Coulter).

### Multiplexed immunofluorescence, tissue imaging, spectral unmixing and phenotyping

A six-color multiplex immunofluorescence panel based on tyramide signal amplification was used for simultaneous detection of CD8 (cytotoxic T lymphocytes), CD4 and Foxp3 (T_regs_), CD11b (myeloid cells), Ki67 (cell proliferative activity), and 4′,6-diamidino-2-phenylindole (DAPI) on tumor sections from formalin-fixed paraffin-embedded (FFPE) samples. The validation pipeline for the multiplex immunofluorescence protocol has been previously described by our group (*82*). At the end of the protocol, nuclei were counterstained with spectral DAPI (Akoya Biosciences) and sections were mounted with Faramount Aqueous Mounting Medium (Dako).

Multiplexed immunofluorescence slides were scanned on the Vectra-Polaris Automated Quantitative Pathology Imaging System (Akoya Biosciences), as described earlier (*82*). Whole tissue present in a single FFPE tissue section was imaged, spectrally unmixed, and exported as component TIF image tile using Akoya Biosciences’ Inform software (version 2.4.8). Component TIF image tiles were then imported into the open-source digital pathology software QuPath version 0.2.0-m9 and stitched together using the *x*-*y* coordinates to create a new pyramidal TIF file for image analysis. Image analysis was performed in the whole tissue sections. Cell segmentation was performed in the whole multispectral image using QuPath software version 0.2.0-m9. Nuclear detection was carried out on the DAPI channel using a custom, unsupervised watershed algorithm as described earlier. A random trees algorithm classifier was generated to further subclassified the cells as CD3^+^, CD8^+^, CD4^+^ Foxp3^+^, CD11b^+^, and Ki67^+^. Cells close to the border of the images were removed to reduce the risk of artifacts. CD4^+^ T cells were defined as CD3^+^ CD8^−^. Cells negative for these markers were defined as “other cell types.” Measurements were calculated as cell densities (cells/mm^2^).

### Assessments of IFN-γ and IL-2 concentrations

Concentrations of IFN-γ or IL-2 in the supernatants of cell cultures were measured by enzyme-linked immunosorbent assay (ELISA) from BD OptEIA (human and mouse IFN-γ ELISA set and human IL-2 ELISA set).

### Statistical analysis

Results were presented as mean ± SD. GraphPad Prism 8 (LA Jolla, CA) was used for appropriate statistical analysis as indicated in the figure legends. Significance is marked on figures as *P < 0.05, **P < 0.01, ***P < 0.001, and ****P < 0.0001.
