# Supplementary material for: CD137 (4-1BB) requires physically associated cIAPs for signal transduction and antitumor effects
Source: Sci Adv. 2023 Aug 18;9(33):eadf6692. doi: 10.1126/sciadv.adf6692 (PMC11044178; doi:10.1126/sciadv.adf6692)
Supplement: Supplementary file 1 — Figs. S1 to S4 Legends for data S1 and S2 [file sciadv.adf6692_sm.pdf]

Science Advances
AAAS

Supplementary Materials for

CD137 (4-1BB) requires physically associated cIAPs for signal transduction and antitumor effects

Javier Glez-Vaz *et al.*

Corresponding author: Ignacio Melero, imelero@unav.es

Sci. Adv. **9**, eadf6692 (2023)
DOI: 10.1126/sciadv.adf6692

The PDF file includes:

Figs. S1 to S4
Legends for data S1 and S2

Other Supplementary Material for this manuscript includes the following:

Data S1 and S2

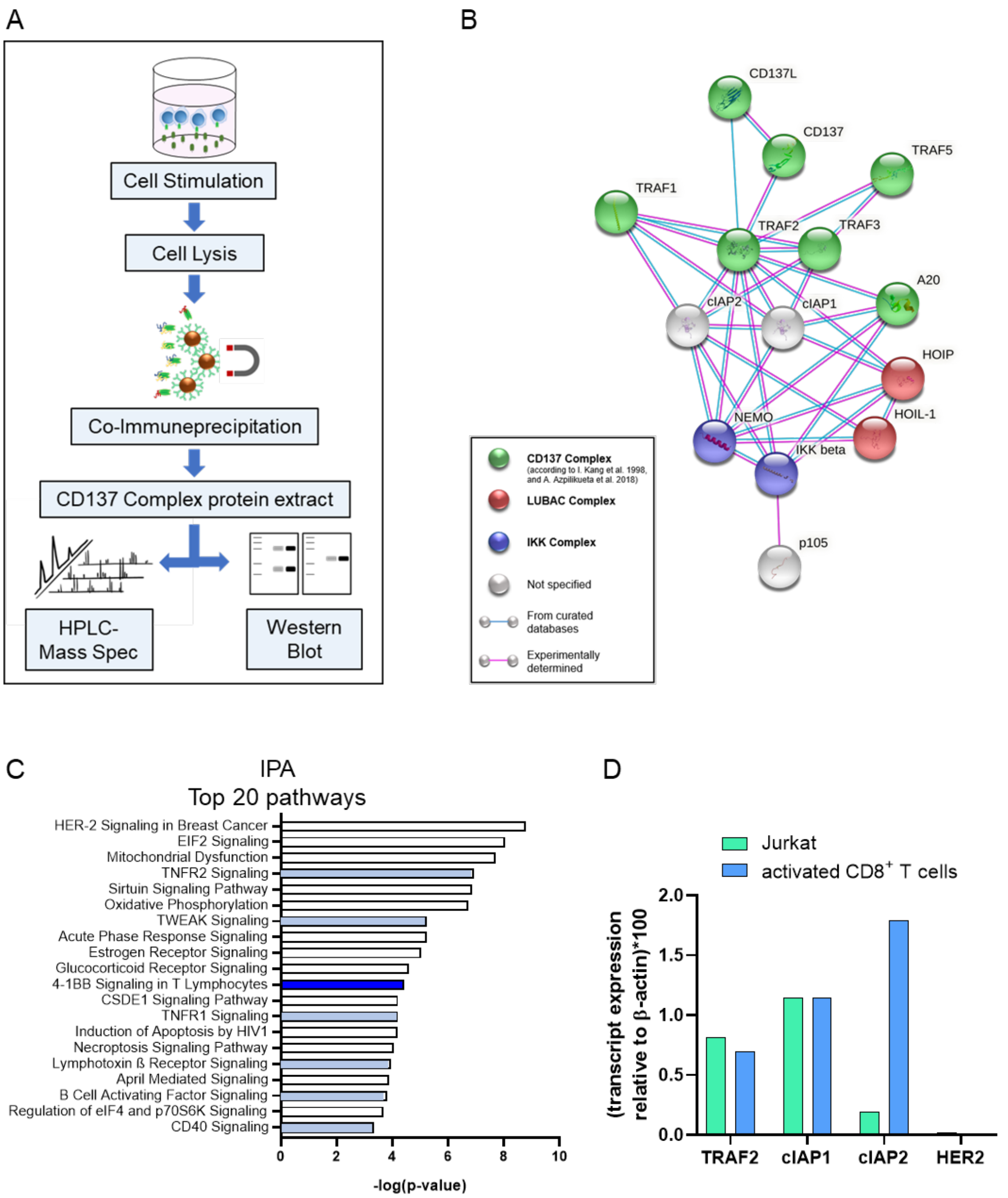

Fig. S1. Co-immunoprecipitation analyses. A) Scheme of the experiments presented in Fig 1. B) Literature-reported protein-to-protein interactions that fit the CD137 co-immunoprecipitation results. C) Functional Ingenuity Pathway Analysis (IPA) of the functions corresponding to the proteins associated to CD137 according to abundance of PSMs that were identified in the human CD8+ T-cell immunoprecipitates. (D) Real-time RT-PCR analysis of the mRNA expression of TRAF2, cIAPs and HER2 in CD137 Jurkat transfectants or primary human CD8 T cells as indicated.

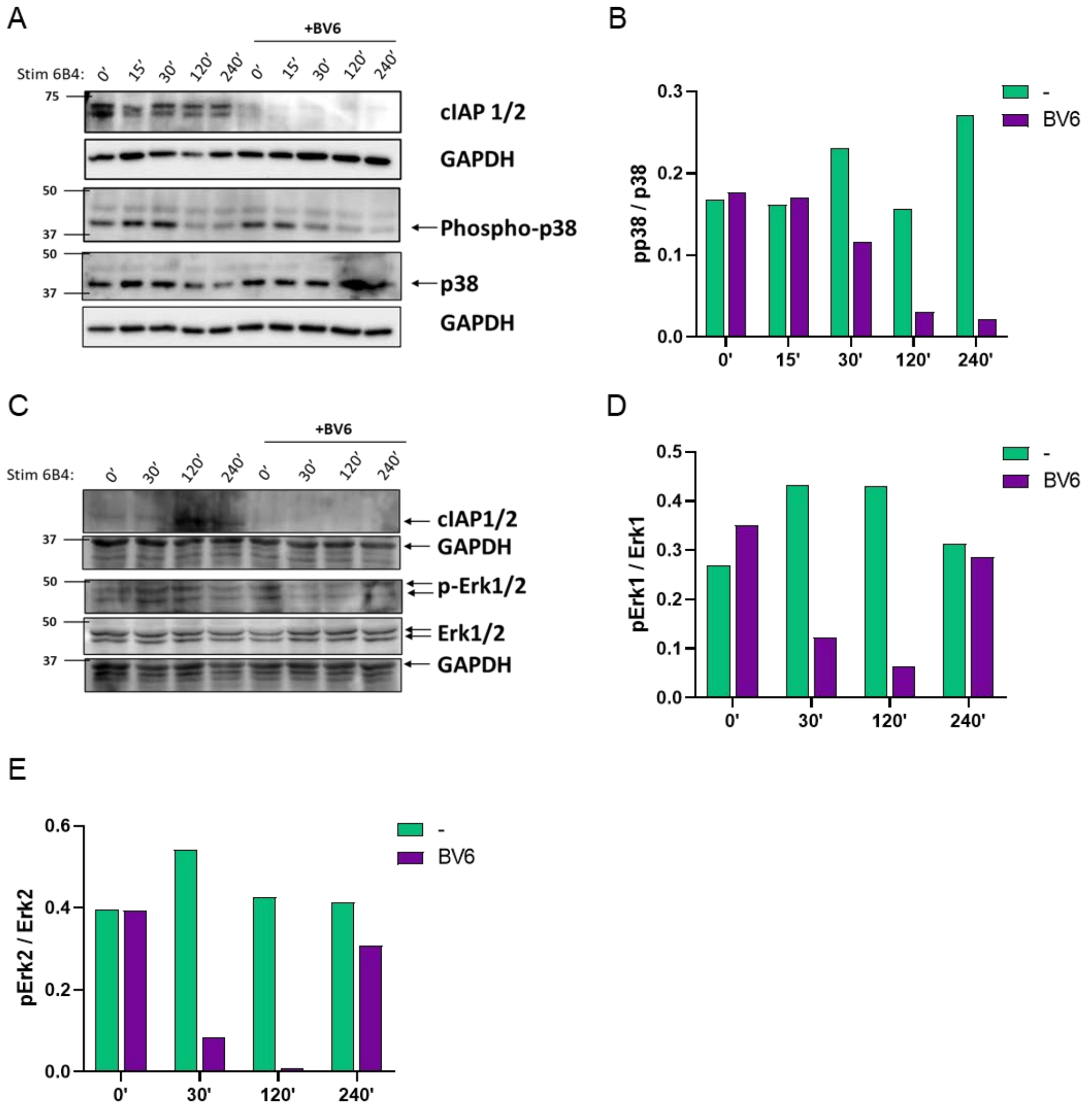

Fig. S2. CD137-elicited MAPK activation is dependent on cIAPs. Human CD8 pre-activated T cells as those in Figure 1D were stimulated with CD137L-Fc attached to the bottom of the plate. (A) Western Blot analyses of cIAP1/2, phosphorylated p38 and total p38. When indicated the SMAC-mimetic BV6 was added to the cultures. Lysates were also immunoblotted to assess the expression of house-keeping GAPDH as a loading control. (B,) shows the densitometry results from A. (C) Similar western-blot analysis of phospho-ERK1/2 in lysates from CD137-transfected Jurkat cells stimulated for the indicated periods of time with soluble anti-CD137 (6B4) mAb. Membranes were also stained for cIAP1/2, total ERK1/2 and GAPDH as a loading control. (D and E) Densitometries corresponding to C.

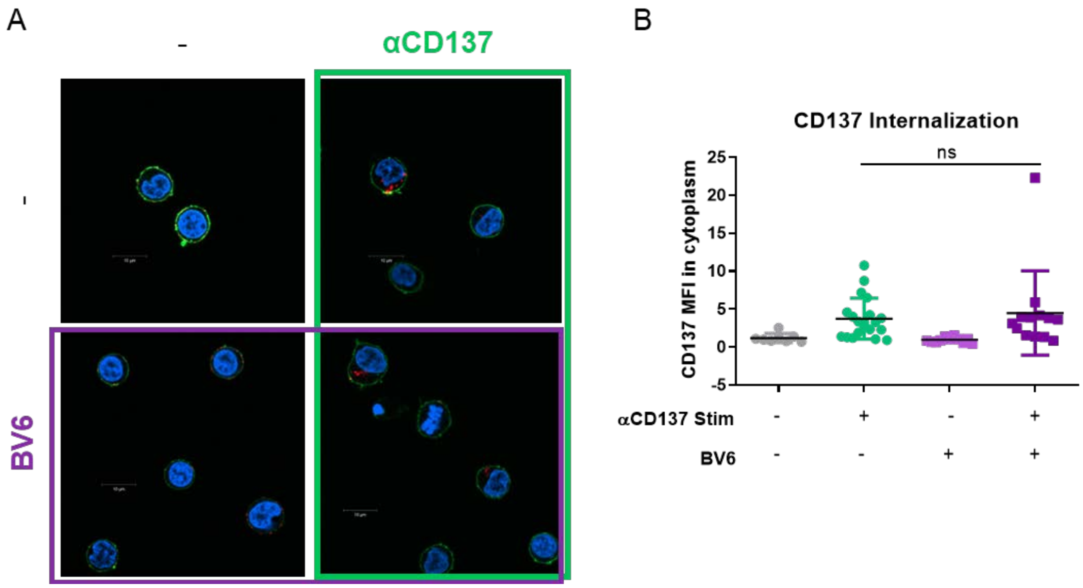

Fig. S3. Lack of effects of SMAC-mimetics on CD137 internalization induced by agonist αCD137 antibodies. (A) Representative confocal microscopy images of internalization following 15 min incubation with Alexa-fluor 647-labelled 6B4 monoclonal antibody and counterstain with HOECHST and WGA-AF488 labelling. When indicated cells had been precultured for 2h in the presence of the SMAC-mimetic BV6. (B) Quantification of the level of internalization measured as relative MFI quantified in the cytoplasm.

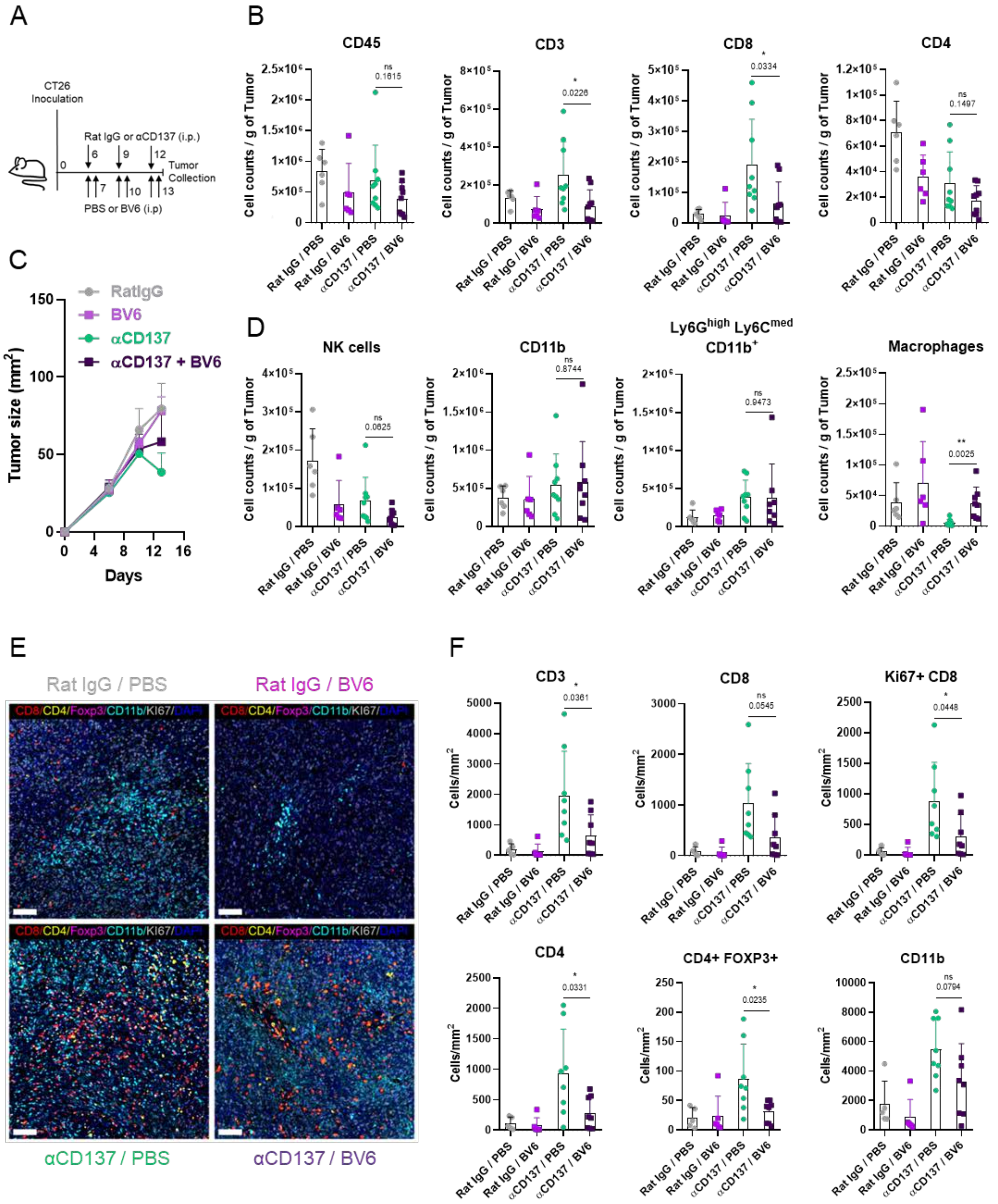

Fig. S4. Increases in tumor T-cell infiltration induced by a CD137 agonist that are markedly reduced by the BV6 Smac mimetic. (A) Schematic representation of the experiments similar to those in Fig. 5A in which tumors were excised on day 13. (B) Assessments in cell suspensions of the density of CD45, CD3, CD8, CD4 leukocytes in the tumors corresponding to the indicated treatment conditions. (C) Average tumor size follow-up until sacrifice and tumor excision (D) Analyses of the density of NK and myeloid leukocytes in the indicated treatment conditions. (E) Representative images of multiplex tissue immunofluorescence stains of tumor sections from the indicated

treatment conditions. (F) Compiled quantitative data of images as in E from individual tumors.

Data S1. (separate file)

Proteins identified in CD137 immunoprecipatates from primary activated human CD8 T lymphocytes.

Data S2. (separate file)

Proteins identified in CD137 immunoprecipatates from a CD137 stable transfectant in Jurkat cells.
